# Drug-related problems and pharmacy interventions in non-prescription medication, with a focus on high-risk over-the-counter medications

**DOI:** 10.1007/s11096-020-00984-8

**Published:** 2020-02-20

**Authors:** Hanna Ylä-Rautio, Sanna Siissalo, Saija Leikola

**Affiliations:** 1 Faculty of Pharmacy, University of Helsinki, Viikinkaari 5 E, P.O. Box 56, 00014 Helsinki, Finland; 2 Association of Finnish Pharmacies, Pieni Roobertinkatu 14 C, 00120 Helsinki, Finland; 3 Pharmac Finland Oy, Rajatorpantie 41 C, 01640 Vantaa, Finland

**Keywords:** Community pharmacy, Drug-related problems, Finland, High-risk medications, OTC, Over-the-counter medication, Pharmacy interventions

## Abstract

*Background* The risks associated with over-the-counter medication are often underestimated by consumers. The incorrect use of certain medications can lead to significant patient harm. Inappropriate use can be prevented by pharmaceutical counselling. *Objective* To determine the number and nature of drug-related problems in over-the-counter medication with a special emphasis on high-risk over-the-counter medications. *Setting* Fifty-two community pharmacies in Finland. *Method* This observational study was conducted as a questionnaire survey. The pharmacists working in participating pharmacies documented the observed drug-related problems and pharmacy interventions in over-the-counter medication during 1 week using an electronic study form based on the Westerlund drug-related problem classification system. *Main outcome measure* The prevalence of drug-related problems and problem types in different medication categories. *Results* The 52 community pharmacies documented 339 drug-related problems in 0.6% of over-the-counter customers, the most common problem being “Uncertainty about the indication for the drug” (39.2%). A significant proportion of the documented problems (26.3%) concerned high-risk over-the-counter medications, and the majority of these cases were associated with non-steroidal anti-inflammatory drugs (21.8%). In total, pharmacies made 641 interventions to resolve the drug-related problems. For majority of drug-related problems (87%), pharmacist’s intervention involved counselling. In more than half of the problem cases, the pharmacy intervention was precautionary. *Conclusion* Pharmacists intervene in and prevent problems related to over-the-counter medications, including high-risk medications like analgesics, in which inappropriate use due to consumers’ lack of knowledge can lead to severe consequences. As the selection and use of over-the-counter medications is continuously increasing, pharmaceutical counselling should be readily available and actively provided for consumers to achieve safer self-medication.

## Impacts on practice

- Many pharmacy customers have inadequate knowledge with regard to the appropriate use of over-the-counter medication.
- Pharmacies recognize and intervene with drug-related problems related to over-the-counter medications; thus, pharmaceutical counselling should be readily available and actively provided in order to prevent drug-related problems.
- The sale of over-the-counter medication outside pharmacies without ensuring sufficient customer counselling is likely to increase the prevalence of adverse outcomes resulting from drug-related problems.
- As a remarkable portion of drug-related problems found was related to systemic analgesics, which are considered as high-risk over-the-counter medications, special emphasis should be given to ensure adequate counselling and safe distribution channels with these medications.

## Introduction

A drug-related problem (DRP) is an event or circumstance involving drug therapy that actually or potentially interferes with desired health outcomes [1]. Pharmacists are highly educated and have a professional responsibility for the efficient counselling of patients to ensure safe use of medications [2]. They play a vital and proactive role in preventing and resolving DRPs [3–5] and thus in preventing adverse events, avoiding extra costs resulting of inappropriate use of medications and adding value to patient safety [6, 7]. Pharmaceutical counselling provided by community pharmacies is particularly crucial when medications are purchased over-the-counter (OTC), without advice given by a physician.

Consumers consider easily accessible OTC medications harmless and thus often underestimate the potential risks [8, 9]. They have an incomplete awareness of several risk areas with regard to OTC medications, such as those relating to drug interactions and misuse/abuse [10, 11]. In a recent Swedish survey on consumers’ views on the safety of OTC medications 7% of respondents agreed completely or to a large extent with the statement that OTCs are completely harmless regardless of how they are being used [12]. An earlier American survey revealed that up to 41% of consumers believe that non-prescription medications are too weak to cause any problems [13]. However, OTC medications can also cause severe harm, and account for adverse drug reaction (ADR)-related hospitalisations, for example. In a German study self-medication accounted for approximately 4% of ADR-related hospitalisations in internal medicine wards; in 53.8% of these cases, the ADRs were due to OTC medications [14].

In Finland, OTC medications, excluding nicotine replacement therapy, can be sold only in pharmacies. OTC medications are usually placed in self-service sections, where a pharmacist is available for advice. Only pharmacists are legitimised to provide medication counselling, but it is possible to purchase OTC medication from a cashier without communicating with a pharmacist e.g. during rush hours or when a customer is reluctant to receive pharmaceutical counselling.

High-risk (high-alert) medications incur a risk of causing significant patient harm when used erroneously [15]. Although most OTC medications are considered to be relatively safe, some can cause serious adverse effects [16, 17]. In Finland, a national high-risk OTC medications list was developed by a group of medication safety experts to prevent severe problems related to OTC medications [18] and has been implemented in practice in a majority of Finnish pharmacies according to the Association of Finnish Pharmacies. In addition to the high-risk medications (non-steroidal anti-inflammatory drugs, paracetamol and potassium), the list includes their key safety risks (e.g. drug–drug interactions) and a check-list to support identification of at-risk patients to prevent DRPs. Pharmacies have been provided with educational material on the usage of the list and encouraged to provide pharmaceutical counselling more actively when selling high-risk OTC medications. To the best of our knowledge, there are no studies on DRPs concerning high-risk OTC medications. Information on the characteristics of DRPs associated with these medications would be beneficial in improving the effectiveness of pharmaceutical counselling and in preventing serious adverse events.

## Aims of the study

The aims of this study were (1) to determine the number and nature of OTC-related DRPs identified by pharmacists in Finnish community pharmacies; (2) to study the prevalence of particular medication categories in the reported cases, with a special emphasis on high-risk OTC medications; and (3) to document pharmacy interventions in the DRP cases.

## Method

### Development of study form

The Westerlund classification system for documenting non-prescription DRPs and pharmacy interventions [19] was applied in this study with slight modifications. The Westerlund system has been used in Swedish community pharmacies in five different versions to document both OTC and prescription medication related DRPs. Modifications to the original Westerlund non-prescription DRP classification were made mainly to collect more information by adding explanatory subclasses (see Table 2). Three classes describing practical problems were united for simplicity, but the original classes were mentioned as examples in the study form. DRP “Drug duplication” was added from a latter version of the Westerlund classification [20].

An electronic study form (Appendix 1) with structured questions to classify the DRPs and pharmacy interventions was developed. In addition, there was an open field for cases in which the pharmacist who filled in the form was uncertain about the accurate classification. Demographic information (gender, estimated age) about the customers was also reported. Information on whether the customer had already used the medication was asked in order to define if the documented intervention was precautionary or corrective.

The classifications and the electronic study form were piloted beforehand in five pharmacies with 33 DRP cases. In the pilot study, pharmacists filled in descriptions of all cases for the classification to be cross-checked by the authors. Based on the experiences from the pilot study, the study form was complemented with additional explanatory instructions to avoid misinterpretations and to support accurate classification.

### Data collection and analysis

This observational study was conducted as a questionnaire survey. The invitation to participate in the study was sent to all members of the Association of Finnish Pharmacies, including their subsidiaries in April 2018 (n = 770 in 2017/95% of all Finnish pharmacy outlets). In the invitation, the pharmacies were provided with written instructions on the DRP classification system and usage of the electronic study form. In addition, it was mentioned that educational material concerning high-risk OTC medications was available online.

**Fig. 1:**
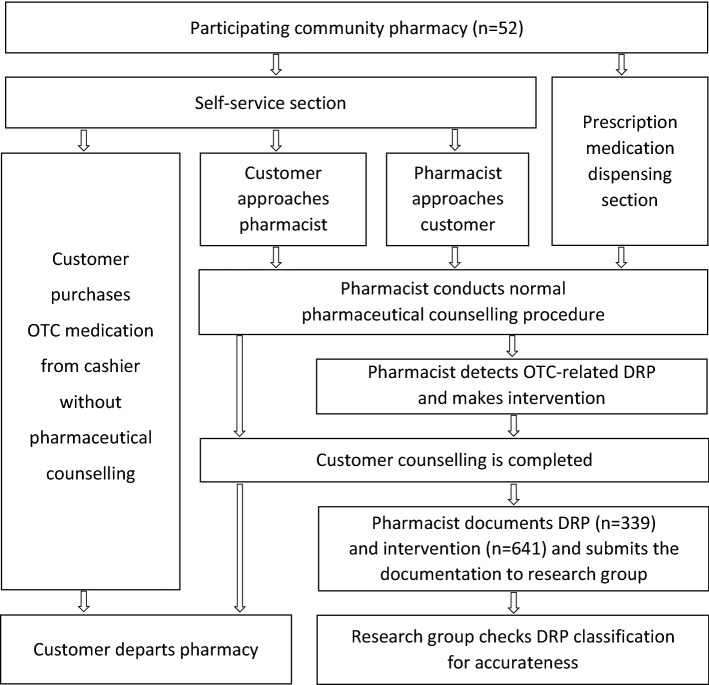
Flow of reporting DRPs and interventions

The participating pharmacies were asked to document all OTC-related DRPs and interventions detected during normal patient counselling for a 1-week time frame. The DRPs were identified during pharmacists’ routine customer counselling; the pharmacists approached and discussed with the customers as they normally do during their work i.e., they did not select any specific customers for the study, neither were they provided with any specific questions to be asked (see Fig. 1). The pharmacists carried out the documentation anonymously after completing counselling to avoid extra inconvenience by using the electronic study form with DRP classification (Appendix 1). During rush hours, the pharmacists could execute the documentation with a paper document and transfer it to the electronic form afterwards. According to the instructions, a new form was to be filled in for each new problem detected. All problems, both potential and actualised, were reported. For each DRP, the pharmacist could document several interventions. If the pharmacist had any hesitations regarding correct classification, an additional open field case description was to be filled in. Furthermore, the number of all customers purchasing OTC medications during the study period was documented, regardless of whether they contacted a pharmacist.

The results were transferred to an Excel spreadsheet and analysed using descriptive statistical methods such as frequencies and percentages. If an open field case description was available, the classification made by the pharmacist was checked for accuracy and reclassified if necessary. In unclear cases the classifications were cross-checked by all authors individually. Differences of opinion were discussed until unanimity was achieved.

The medications reported were classified according to the ATC (Anatomical, Therapeutic, Chemical) classification index level 5 [21]. In addition to the ATC classification, OTC high-risk medications (non-steroidal anti-inflammatory drugs, paracetamol and potassium) [18] were categorised separately (see Table 4).

## Ethics approval

The DRPs were documented anonymously. No personal information regarding the customers, apart from gender and estimated age, were documented. The individual pharmacists could not be identified. Thus, institutional review board approval was unnecessary.

## Results

### Study participants

Initially, 67 pharmacies registered to participate in the study. Eventually, DRP documentation was received from 52 pharmacies (6.4% of all Finnish pharmacies) in May–June 2018 (Table 1). The participating pharmacies were a fairly representative sample of different sizes of pharmacies in Finland, with only the medium-sized pharmacies being slightly overrepresented. In five pharmacies (9.6% of the participating pharmacies) the study period was other than the recommended 1 week (4–14 days) due to practical reasons. The pharmacies documented on average 6.5 DRPs (1–21/pharmacy) and a total of 339 DRPs among 55,296 customers purchasing OTC medication with or without pharmaceutical counselling (0.61% of all OTC customers). The DRP detection rate varied from 0.06% to 2.51% between pharmacies.

**Table 1:** Basic study data

	n	%
Community pharmacies in Finland	812	100
Pharmacies registered to participate	67	8.3
Pharmacies reporting DRPs	52	6.4
Customers purchasing OTC medication in total	55,296	100
DRPs documented	339	0.6
Estimated age (y) of customer		
0–24	27	8.0
25–44	78	23.0
45–64	85	25.1
65–75	106	31.3
> 75	39	11.5
Unknown	4	1.2
Problem detected in		
A self-service section	289	85.3
While dispensing a prescription medication	48	14.2
Unknown	2	0.6
Type of pharmacy intervention		
Precautionary	190	56.0
Corrective	129	38.1
Unknown	20	5.9
Type of product documented		
Medication	297	83.8
Natural or supplementary product	16	8.6
Unknown	26	7.7

### Study population

The two most frequently reported age groups of customers with DRPs were 65–74 years (31.3%) and 45–64 years (25.1%). The majority of the cases were precautionary (56.0%; n = 190), yet in 38.1% of the DRP cases the customer had already used the medication erroneously or in a suboptimal manner. In 20 cases the pharmacist did not receive or document information on whether the customer had already used the medication. The DRPs were usually detected in the self-service sections (85.3%), although 14.2% of the DRPs related to OTC medications were detected while dispensing a prescription medication.

### Drug-related problems

“Uncertainty about the indication for the drug” (39.2%) was the most commonly documented DRP (Table 2). Within this class, the most frequently occurred subclass was “False indication for the drug use”, e.g. long-term use of vasoconstrictive nasal therapy for allergy or for dry nasal mucosa. Second most common problem type was “Overuse of medication” (14.7%) in which the problems were mainly associated with prolonged use of medication. “Drug-drug interactions” (13.0%) were mostly related to prescription medications. Of the documented DRPs, 83.8% (n = 284) were related to medications (Table 1). Natural or supplementary products occurred in 8.6% (n = 29) of the DRPs and in 7.7% (n = 26) of the DRPs the product type was unknown.

**Table 2:** Drug-related problems (DRPs)

DRP	n	%
**Uncertainty about the indication for the drug**	**133**	**39.2**
False indication for the drug use	116	34.2
No indication for the drug use	13	3.8
Intentional misuse of the drug	4	1.2
**Overuse of medication**	**50**	**14.7**
Dosage too high	11	3.2
Duration of drug use too long	39	11.5
**Drug-drug interaction**	**44**	**13.0**
Drug-drug interaction with a prescription drug	37	10.9
Drug-drug interaction with an OTC drug	7	2.1
**Drug duplication**	**33**	**9.7**
**Contraindication**	**20**	**5.9**
**Insufficient drug efficacy**	**19**	**5.6**
**Adverse drug reaction**	**10**	**2.9**
**Underuse of medication**	**9**	**2.7**
Dosage too low	5	1.5
Duration of drug use too short	4	1.2
**Language deficiency**	**5**	**1.5**
**Practical problems**^a^	**3**	**0.9**
**Other dosage problems**^b^	**2**	**0.6**
**Other DRP**	**11**	**3.2**
Total	339	100.0

^a^Including DRPs “Difficulty swallowing tablet/capsule”, “Difficulty opening drug container” and “Other practical problem”

^b^A problem with dosage other than dosage too high or low e.g. taking tablets at wrong time of the day or wrong intake in relation to food

#### Distribution of DRPs according to the ATC classification system

Nearly one-third (31.3%) of all DRPs were related to medications affecting the respiratory organs (ATC class R) (Table 3). Of these, vasoconstrictive nasal therapy accounted for 16.8% (n = 57) of all DRPs, “False indication for the drug use” (n = 30; 8.8%) and “Duration of drug use too long” (n = 24; 7.1%) being the most frequently occurring problems. In subclass R06 (antihistamines for systemic use), the vast majority of the documented problems concerned treatment of an allergy (n = 27), of which the most common issue was “Insufficient drug efficacy” (n = 11).

**Table 3:** Number of DRPs according to the ATC classification system; five most frequently reported main classes and three subclasses

ATC class		n	%
*R*	*Respiratory organs*	106	31.3
R01	Nasal preparations	58	17.1
R06	Antihistamines for systemic use	30	8.8
R05	Cough and cold preparations	15	4.4
*M*	*Musculo-skeletal system*	49	14.5
M01	Anti-inflammatory and anti-rheumatic products	46	13.6
M02	Topical products for joint and muscular pain	3	0.9
*A*	*Alimentary tract and metabolism*	47	13.9
A07	Antidiarrheals, intestinal anti-inflammatory/anti-infective agents	13	3.8
A02	Drugs for acid-related disorders	12	3.5
A12	Mineral supplements	9	2.7
*N*	*Nervous system*	40	11.8
N02	Analgesics	36	10.6
N05	Psycholeptics	2	0.6
N07	Other nervous system drugs	2	0.6
*D*	*Dermatologicals*	22	6.5
D07	Corticosteroids, dermatological preparations	10	2.9
D03	Preparations for treatment of wounds and ulcers	5	1.5
D01	Antifungals for dermatological use	3	0.9
	Total	264	77.9

ATC class A (alimentary system) accounted for nearly 14% of all DRPs. With antidiarrheals the most frequently reported problem was “False indication for the drug use” and with drugs for acid-related disorders the most common issue was “Duration of drug use too long”. In ATC class D (dermatologicals) preparations containing hydrocortisone (n = 10) or dexpanthenol (n = 5) accounted for the majority of the documented DRPs. With dermatologicals, “False indication for the drug use” (n = 19) was the most common problem type.

#### High-risk OTC medication

The majority of documented problems in ATC class M (musculoskeletal system) were related to ibuprofen (n = 42), which is classified as a high-risk OTC medication. Furthermore, the high-risk OTC medications acetylsalicylic acid (ASA) (n = 23) and paracetamol (n = 13) accounted for the majority of the problems in ATC class N (nervous system). Depending on the indication, ASA is also classified in ATC class B (blood and blood forming organs) in which seven ASA-related problems were reported.

Overall, a significant proportion of the documented problems (26.3%) concerned high-risk OTC medications (Table 4). The majority of these cases were associated with NSAIDs (21.8%) in which the most common issues were “Drug-drug interaction with a prescription drug” (n = 22) and “Inappropriate duplication of therapeutic group or active ingredient” (n = 21). In addition to the previously mentioned problems, “No indication for the drug use” was reported in six ASA cases concerning mainly low-dose use as an antithrombotic agent without consulting a physician first. Paracetamol-related problems (3.8% of all DRPs) were mainly associated with unintentional overuse.

**Table 4:** DRPs related to high-risk OTC medication [18]

ATC class	Medication	n	%	Most frequent problems (n)
M01AE01	Ibuprofen	42	12.4	Drug-drug interaction with a prescription drug (12). Inappropriate duplication of therapeutic group or active ingredient (9). False indication for the drug use (8)
B01AC06. N02BA01. N02BA51	Acetylsalicylic acid (ASA)	30	8.8	Inappropriate duplication of therapeutic group or active ingredient (10). Drug-drug interaction with a prescription drug (10). No indication for the drug use (6)
N02BE01	Paracetamol (acetaminophen)	13	3.8	Inappropriate duplication of therapeutic group or active ingredient (4). Dosage too high (3). False indication for the drug use (2)
M01AE03	Ketoprofen	2	0.6	Inappropriate duplication of therapeutic group or active ingredient (2)
A12BA01	Potassium chloride	2	0.6	False indication for the drug use (1). Language deficiency (1)
	Total	89	26.3	

#### Vitamins and natural medications or other products

Vitamins and natural medications or other products appeared in less than 9% of the DRP cases (Table 1). The most frequently reported product category was lactic acid bacteria preparations (n = 3), otherwise the reported problems occurred in individual products only. “False indication for the drug use” (n = 8) was also the most common problem in this category.

### Number and types of pharmacy interventions

In total, pharmacies made 641 interventions (1–5 interventions/DRP, on average 1.9 interventions/DRP; Table 5). The most common types of pharmacy interventions were “Customer/representative drug counselling” (87.0% of DRPs) and “Switch of drug” (51.3% of DRPs). In one-fifth of the DRP cases, the pharmacist recommended referral to a physician and in one-fifth refused to sell the medication. In addition, non-medical treatment was recommended in 6.2% of the cases.

**Table 5:** Pharmacy interventions

Intervention	n	%^a^
Customer/representative drug counselling	295	87.0
Switch of drug	174	51.3
Referral to physician	73	21.5
Refusal to sell the drug	71	20.9
Recommended treatment without drugs	21	6.2
Other intervention	6	1.8
No intervention	1	0.3
Total	641	100.0

More than one intervention could be selected for one DRP

^a^Percentage of the reported problems (n = 339)

## Discussion

### DRP detection rate

In our study, DRPs were documented in 0.6% of all OTC customers. However, there was a more than 40-fold variation between pharmacies in the DRP detection rate. Although similar results have been obtained earlier [19], the DRP prevalence is surprisingly low considering our experiences of everyday practice in community pharmacies. The low detection rate may be due to not identifying all of the problems, or not documenting each problem detected. Furthermore, especially in more crowded pharmacies and during busy hours, it may not be possible for the pharmacist to reach all customers for discussion and counselling. This issue of under-detection has been recognized as a common limitation for studies approaching DRP detection [22]. In our study, the number of DRPs was compared to the number of all OTC customers, regardless of whether they had any contact with a pharmacist. The DRP detection rate may also be influenced by differences in activity between individual pharmacists.

### DRP types

In the reported DRP cases, the most common problems were “Uncertainty about the indication for the drug” and “Overuse of medication”, somewhat similar to the earlier studies [4, 19, 23]. Unexpectedly, “Drug-drug interaction” was found in 13.0% of the DRP cases, whereas previously interactions have accounted for only 3.0–4.1% of the DRPs [19, 23]. Since the most common OTC medications affected by drug–drug interactions in Finland are NSAIDs [24], the recent implementation of the high-risk list including this interaction potential may have increased the pharmacists’ awareness of the potentially harmful interactions, as well as encouraged them to intervene.

The prevalence of intentional misuse was rather low in our study. The intentional misuse is hard to recognize since most patients endeavour to hide it; thus, it may have been underestimated. However, the frequently occurred inappropriate use of vasoconstrictive nasal therapy could be regarded as intentional misuse in some cases. Although usually started for genuine symptoms, the incorrect use was often continued even though customers had been informed several times about the risks according to the open-field case descriptions. Inappropriate use of these medications is rarely fatal, but it can result in increasing tolerance and have undesirable effects on the mucosa [25].

### Pharmacy interventions

Most of the detected DRPs could be effectively managed in the pharmacies; pharmacy interventions “Customer/representative drug counselling” and “Switch of drug” accounted for 73.2% of the interventions (73.3% in a previous study [19]). However, pharmacists also referred patients to the physician when needed or refused to sell the inappropriate medication; both of these accounted for over one-fifth of the DRP cases. In Finland, ethics is considered highly important in pharmaceutical practice. According to the Ethical Guidelines for Community Pharmacy Practice, patients’ best interests must be preferred over business aspects. The high prevalence of interventions “Referral to physician” and “Refusal to sell the drug” suggest that these Guidelines indeed are followed by pharmacists.

In more than half of the DRP cases, the intervention was precautionary; the patient had not used the medication erroneously yet. This is a remarkable finding since by preventing DRPs, potential adverse drug events (ADEs) may be avoided [7]. Hence, pharmaceutical counselling contributes to both reducing health-care costs and improving quality of life for individual patients. However, inappropriate use of a medication does not always lead to harmful consequences. More studies are needed to estimate the impact of interventions on health outcomes and health-care costs.

### DRPs related to high-risk OTC medications

A significant proportion of the detected problems in our study concerned high-risk OTC medications; NSAIDs and paracetamol accounted for over 25% of the DRPs. This is not surprising as ibuprofen and paracetamol are the two most frequently sold OTC medications in Finland [26]. With these medications, the pharmacists’ role in preventing DRPs is particularly important since incorrect use could lead to serious consequences, such as harmful drug–drug interactions, hospitalisations and even death, especially among the polymedicated elderly and other risk groups [14, 24, 27–29]. Pharmacists are likely to be aware of serious ADEs, and the newly developed OTC-risk list has also increased their awareness of the potential harms. The high pharmacist intervention rate in our study is a positive finding, as several studies indicate that consumers have gaps in their knowledge when it comes to the correct use of NSAIDs and paracetamol [30–33]. Misunderstanding the active ingredient, maximum daily dose, contraindications and potential side effects may be placing consumers at risk of experiencing serious ADEs [30]. Furthermore, simultaneous use of several products with a different brand name is common and could result in overdosing [33]. Overdosing with paracetamol in particular can lead to severe outcomes, although it is otherwise considered to be well-tolerated and safe also among more vulnerable patients such as children and the elderly.

In Finland, the risks associated with inappropriate use of medications have been taken seriously since most OTC medications are only available in pharmacies. Nevertheless, there is persistent political debate about releasing OTC medications for sale outside pharmacies. Our study indicates that high-risk OTC medications in particular are unsuitable for liberalisation; without pharmaceutical counselling, DRPs could neither be prevented nor corrected. For example, in Sweden, the incidence of paracetamol poisoning increased after it was made available in non-pharmacy outlets in 2009 [34]. As a result, paracetamol tablets were reclassified and have only been available in pharmacies since 2015.

### Limitations and strengths

In terms of limitations, the participation rate in this study was rather low. Nevertheless, the participating pharmacies constituted a fairly representative sample of different sizes of pharmacies in Finland. Secondly, although in line with a previous study [19], the fairly low DRP prevalence suggests that all occurring problems were not documented—or not identified—which may have affected the DRP distribution in different medication categories. The DRP prevalence altogether is also affected by the presence and approachability of pharmacists in the OTC section. In addition, there may have been misunderstandings concerning the correct classification, and some variation between pharmacists. However, the classification of the DRPs and interventions was carefully piloted beforehand and complemented with explanatory instructions and an open field for unclear cases to avoid misinterpretation. Thirdly, there is seasonal fluctuation in the consumption of medications. As the study was conducted in spring, it may have resulted in both over- and underestimation of certain groups of medications. Medications for allergy treatment may have been overrepresented, whereas the prevalence of analgesics might be even higher during the influenza season in winter.

As a strength in this study, a validated DRP classification system, which has been used in several previous studies, was applied. This improves comparability and validity of our results. In addition, the classification was carefully piloted beforehand. In order to increase reliability of the classification, a possibility to fill in an open field case description was provided. These descriptions were cross-checked by the authors and reclassified if needed. To achieve as high documentation rate as possible the documentation process was designed effortless by using both the electronic study form and the additional paper document.

## Conclusion

This study indicates that pharmacists intervene in and prevent problems related to high-risk OTC medications, especially analgesics. In this particular medication group, inappropriate use due to consumers’ lack of knowledge may lead to severe consequences. As the selection and use of OTC medications is continuously increasing, pharmaceutical counselling should be readily available and actively provided for consumers in order to achieve safer self-medication.
